# Modelling the impacts of pasture contamination and stocking rate for the development of targeted selective treatment strategies for *Ostertagia ostertagi* infection in calves

**DOI:** 10.1016/j.vetpar.2017.03.025

**Published:** 2017-04-30

**Authors:** Zoe Berk, Yan C.S.M. Laurenson, Andrew B. Forbes, Ilias Kyriazakis

**Affiliations:** a School of Agriculture Food and Rural Development, Newcastle University, Newcastle upon Tyne, NE1 7RU, UK; b Animal Science, School of Environmental and Rural Science, University of New England, Armidale, New South Wales 2351, Australia; c Scottish Centre for Production Animal Health and Food Safety, School of Veterinary Medicine, University of Glasgow, G61 1QH, Scotland, UK

**Keywords:** Cattle, Gastrointestinal parasitism, *Ostertagia ostertagi*, Targeted selective treatment (TST), Anthelmintic resistance, Stocking rate

## Abstract

A simulation study was carried out to assess whether variation in pasture contamination or stocking rate impact upon the optimal design of targeted selective treatment (TST) strategies. Two methods of TST implementation were considered: 1) treatment of a fixed percentage of a herd according to a given phenotypic trait, or 2) treatment of individuals that exceeded a threshold value for a given phenotypic trait. Four phenotypic traits, on which to base treatment were considered: 1) average daily bodyweight gain, 2) faecal egg count, 3) plasma pepsinogen, or 4) random selection. Each implementation method (fixed percentage or threshold treatment) and determinant criteria (phenotypic trait) was assessed in terms of benefit per R (BPR), the ratio of average benefit in weight gain to change in frequency of resistance alleles R (relative to an untreated population). The impact of pasture contamination on optimal TST strategy design was investigated by setting the initial pasture contamination to 100, 200 or 500 *O. ostertagi* L3/kg DM herbage; stocking rate was investigated at a low (3calves/ha), conventional (5 calves/ha) or high (7 calves/ha) stocking rates. When treating a fixed percentage of the herd, treatments according to plasma pepsinogen or random selection were identified as the most beneficial (i.e. resulted in the greatest BPR) for all levels of initial pasture contamination and all stocking rates. Conversely when treatments were administered according to threshold values ADG was most beneficial, and was identified as the best TST strategy (i.e. resulted in the greatest overall BPR) for all levels of initial pasture contamination and all stocking rates.

## Highlights

• Stocking rate effect on design of targeted selective treatments (TST) was evaluated.
• Initial pasture contamination effect on the design of TST was evaluated.
• Different phenotypic traits and methods of selection for treatment were addressed.
• Benefit was assessed as weight gain/frequency of resistant alleles in helminths.
• Treatment according to threshold triggers of average daily gain was most beneficial.

## 1 Introduction

Targeted selective treatment (TST) can prolong effective parasite control in livestock systems reliant upon anthelmintic drugs (van Wyk et al., 2006) by only treating individual animals with a high parasitic burden or poor parasite tolerance. Untreated individuals continue to contribute susceptible parasite genotypes to pasture (i.e. increasing *refugia*) which reduces selection pressure for drug resistance and thereby prolongs anthelmintic efficacy. Two methods have previously been proposed for implementing TST strategies: 1) treatment of a fixed percentage of a herd according to a given phenotypic trait (Laurenson et al., 2013), or 2) treatment of individuals that exceed a threshold value for a given phenotypic trait (Charlier et al., 2014). Experimental studies on TST in cattle have previously investigated the use of various phenotypic traits as determinant criteria for treatment. These include performance traits, such as average daily bodyweight gain (ADG, kg/d) (Höglund et al., 2013), and parasitic traits, such as faecal egg count (FEC, eggs/g) and plasma pepsinogen (O’Shaughnessy et al., 2014).

To date no experimental studies have been conducted to directly compare the methods for implementing TST strategies or the phenotypic traits used as determinant criteria for treatment. However, previous simulation studies have made such comparisons by determining the consequent benefit per R (BPR, kg/R) (Berk et al., 2016c, Laurenson et al., 2016). BPR is calculated as the ratio of the average benefit in weight gain resulting from treatment, relative to the frequency of drug resistance alleles (R) amongst parasites resulting from treatment. BPR can thereby identify the selection method and phenotypic trait resulting in the greatest productive gain per impact upon anthelmintic efficacy. As such, a previous simulation study (Berk et al., 2016c) concluded that treatment according to threshold values of ADG was most beneficial (i.e. resulted in the greatest BPR) for a conventional stocking rate of 5 calves/ha (AHDB, 2013) and an initial pasture contamination (IL_0_) of 200 infective L3 larvae/kg DM (Larsson et al., 2007). However, variation in IL_0_ or stocking rate have previously been demonstrated to have significant consequences upon parasitological and epidemiological outputs (Berk et al., 2016a); and may therefore be expected to heavily influence the build-up of resistance. Hence, the aim of the current simulation study was to evaluate whether variations in IL_0_ or stocking rate had a significant effect either on the best selection method for TST or the best determinant criterion (phenotypic trait) on which to base treatment, as assessed by BPR.

## 2 Material and methods

The simulation model of Berk et al. (2016c) was used as the basis of this study. The model describes the impacts of *O. ostertagi* on a population of growing calves, taking into account variation in host phenotypes, host-parasite interactions, parasite epidemiology and anthelmintic resistance amongst nematode populations. As described by Berk et al. (2016b), between-animal phenotypic variation was included in calf growth attributes (optimal growth rate and body composition at maturity), maintenance requirements, and the ability to mount an immune response (rate of acquisition, as well as initial and final rates for host-controlled establishment, mortality and fecundity). Simulations were based on a herd of weaned, castrated male (steer) Limousin × Holstein Friesians, a common cross of beef cattle reared in the UK (Todd et al., 2011). It was assumed the calves were autumn-born and capable of utilising grass in early spring; hence calves were turned out at 6 months of age and left on pasture for a further 6 months until housing in late autumn (Phillips, 2010). A population of 500 calves was simulated over the first grazing season, with the same population being modelled for all treatment groups. This population size was chosen to predict population means with sufficient accuracy to allow statistical comparison between treatment groups. Smaller and more typical herd sizes could be simulated by the model; however, in that case it would be necessary to perform multiple simulations to obtain a proper statistical description of the herd characteristics (Berk et al., 2016b). All calves were assumed to have no prior exposure to parasites. An initial pasture contamination was assumed to arise as a result of overwintered eggs and larvae, and the subsequent larval contamination of pasture was assumed to be a consequence of eggs excreted by infected calves. Anthelmintic resistance was assumed to be controlled by 2 genes, assuming perfect gene and allele neutrality. Anthelmintic efficacy against susceptible nematodes was defined according to Yazwinski et al. (2009) and assumed to exhibit a sigmoidal decay over time. The initial frequency of the recessive alleles conveying anthelmintic resistance (R) was assumed to be 0.001 on pasture (Barnes and Dobson, 1990). The frequency of each genotype over time was calculated using the Hardy-Weinberg equilibria and all genotypes were assumed to be equally fit on pasture.

All model simulations were programmed in MATLAB (2015). In the current study this model was used to evaluate whether variations in IL_0_ or stocking rate impacted upon recommendations in regards to the best selection method (fixed percentage or threshold treatments) and determinant criterion (phenotypic trait) for use in a TST strategy.

### 2.1 TST based on fixed herd percentages

A previous study (Berk et al., 2016c) predicted that the frequency of R on pasture increased exponentially with the percentage of calves treated. This resulted in a decreasing BPR for increasing percentages of the calves treated, meaning that the rapid build-up in frequency of R was not justified by the accompanying improvement in weight gain. For this reason, the current study only investigated the consequences of treating 10% or 25% of the host population. Individual calves were assessed for treatment at 8 and 16 weeks post-turnout. Three determinant criteria for treatment were investigated; ADG, FEC and pepsinogen (international units of tyrosine/litre, modelled as a function of worm burden (Berk et al., 2016c)). These determinant criteria were selected as good indicators of parasite load, or resulting compromised performance. As such, calves were preferentially treated according to lowest ADG, highest FEC or highest pepsinogen level. An additional comparison group was included whereby a fixed percentage of calves were selected for treatment at random; the relative success of each determinant criterion was subsequently evaluated in contrast to this.

### 2.2 TST based on threshold values

For treatments triggered by threshold values, the same three determinant criteria of ADG, FEC and pepsinogen were investigated. Assessment for treatment was made every 3 weeks from 8 weeks post-turnout by which point parasitic burdens and pasture contamination began to increase and bodyweight gains were compromised (Höglund et al., 2013, O’Shaughnessy et al., 2015). When ADG was used as the determinant criterion, a threshold value was calculated on the basis of the average ADG taken for the poorest growing 50% of the population of strategically treated calves (whole-herd treatment at 3, 8 and 13 weeks). This was assumed to provide conservative estimates of the expected ADG in a healthy population; calves were treated when individual ADG was inferior to this threshold value. A FEC threshold value of 80 *O. ostertagi* eggs/g was assumed, based on a previously investigated threshold of 200 eggs/g for mixed infections (O’Shaughnessy et al., 2014) and assuming *O. ostertagi* eggs comprise a proportion of 0.4 (Ploeger and Kloosterman, 1993). Individuals displaying FECs above this were treated with anthelmintic. Populations treated according to threshold values for plasma pepsinogen were treated when levels exceeded 2 IUT/l (O’Shaughnessy et al., 2015).

### 2.3 Simulation procedures

#### 2.3.1 Varying initial pasture contamination

To investigate the impact of IL_0_, starting values of 100, 200 or 500 *O. ostertagi* L3/kg DM (Berk et al., 2016b) were simulated, and the grazing area was set to 100 ha representing a conventional stocking rate of 5 calves/ha (AHDB, 2013). These values were selected to cover the range of possible IL_0_ levels that are likely to occur (Shaw et al., 1998). When treating a fixed percentage of calves, a 3 × 4 × 2 factorial design was used to compare the effects of IL_0_ level, determinant criteria and the percentage of calves treated. When treating calves according to threshold values a 3 × 3 factorial design was used to compare the effects of IL_0_ and determinant criteria.

#### 2.3.2 Varying stocking rate

To investigate TST strategies under differing stocking rates, IL_0_ was set to 200 *O. ostertagi* L3/kg DM, and the grazing area adjusted for low (3 calves/ha), conventional (5 calves/ha) and high (7 calves/ha) stocking rates as defined by AHDB (2013). When treating a fixed percentage of calves a 3 × 4 × 2 factorial design was used to compare the effects of stocking rate, determinant criteria and the percentage of calves treated. When treating calves according to threshold values a 3 × 3 factorial design was used to compare the effects of stocking rate and determinant criteria.

### 2.4 Simulation outputs

In order to evaluate each of the simulated strategies, the ‘benefit per R’ (BPR) was calculated to account for production benefits and the impact on anthelmintic resistance such that equal weighting was given to both traits. BPR at time of housing was calculated according to Laurenson et al. (2016) as follows:

(1) $${BPR}_{h}=\frac{{EBW}_{{TST}_{h}}-{EBW}_{C_{h}}}{{RAF}_{{TST}_{h}}-{RAF}_{C_{h}}}$$

where ${EBW}_{{TST}_{h}}$ is the empty body weight (kg) at the time of housing (*h*) for a group of calves receiving a given TST strategy, ${EBW}_{C_{h}}$ is the empty body weight (kg) at time of housing for a group of calves left untreated, ${RAF}_{{TST}_{h}}$ is the frequency of the R allele on pasture at time of housing (*h*) for a group of calves receiving a given TST strategy and ${RAF}_{C_{h}}$ is the frequency of the R allele on pasture at time of housing for a group of calves left untreated.

The BPR output was a single measure for the complete pasture and therefore variation was estimated by simulating 10 populations for each treatment group. For the TST strategies based on fixed herd percentages a two-tailed Z test comparison of BPR was conducted to assess whether determinant criteria showed a significantly different outcome to random selection (assessed at 95% confidence intervals). Upon comparing threshold treatments no statistical comparison was made for lack of a suitable standard control group.

## 3 Results

Berk et al. (2016c) previously described model predictions for: average worm burden, pasture contamination (L3/kg DM), frequency of R on pasture, FEC (eggs/g DM) and the relative reduction in empty body weight gain (kg in comparison to a non-parasitized herd) for fixed percentage and threshold treatments administered according to ADG, FEC, pepsinogen or random selection for a conventional stocking rate of 5 calves/ha and an IL_0_ of 200L3/kg DM. In the current study, only BPR predictions are detailed as this was the trait against which each of the simulated strategies were ultimately evaluated.

### 3.1 TST based on fixed percentages

#### 3.1.1 Varying initial pasture contamination

**Fig. 1:**
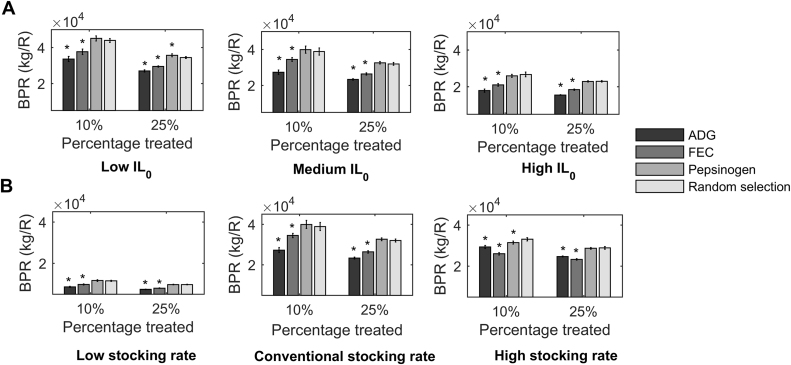
End of season (day 180) predictions for benefit per R (BPR), representing the benefit in empty bodyweight gain (kg) per change in frequency of resistant alleles amongst parasites (R), so the higher the value the more beneficial the strategy is perceived to be. Predictions are provided as an average of ten discrete populations of 500 calves simulated calves grazing on A) pasture initially contaminated with 100, 200 and 500 L3/kg DM grass at a conventional stocking rate of 5 calves/ha and B) pasture initially contaminated with 200 L3/kg DM grass for calves kept at stocking rates of 3, 5 and 7 calves/ha. Anthelmintic treatment was administered at weeks 8 and 16 to either 10 or 25% of the population according to lowest average daily bodyweight gain (ADG, kg/d), highest faecal egg count (FEC, eggs/g), highest pepsinogen (IUT/l) or selected at random. (* p < 0.05).

Fig. 1A provides the BPR (kg/R) predictions for all levels of IL_0_ when fixed percentages (10% or 25%) of the simulated herd were treated according to the determinant criteria of ADG, FEC, pepsinogen or random selection. For all levels of IL_0_ and each determinant criterion, reducing the percentage of the herd treated led to an increased BPR. For both fixed treatment percentages and each determinant criteria, reducing IL_0_ led to an increased BPR. For all IL_0_ and both fixed treatment percentages, the determinant criteria of ADG and FEC resulted in significantly worse BPR predictions than random selection. Using pepsinogen as the determinant criterion was predicted to result in significantly better BPR predictions than random selection when 25% of the herd was treated at low IL_0_; however, BPR predictions for pepsinogen were not significantly better than random selection for the medium and high IL_0_.

#### 3.1.2 Varying stocking rate

Fig. 1B provides the BPR (kg/R) predictions for all stocking rate levels when a fixed percentage (10% or 25%) of the simulated herd were treated according to the determinant criteria of ADG, FEC, pepsinogen or random selection. For all stocking rates and each determinant criterion, reducing the percentage of the herd treated led to an increased BPR. For both fixed treatment percentages and each determinant criterion, BPR predictions were greatest for the conventional stocking rate, closely followed by the high stocking rate, with BPR being greatly reduced for the low stocking rate. For all stocking rates and both fixed treatment percentages, the determinant criteria of ADG and FEC resulted in significantly worse BPR predictions than random selection. BPR predictions for pepsinogen were not significantly better than random selection for all stocking rates or either fixed treatment percentages, with the exception of the high stocking rate when 10% of the herd were treated, where BPR predictions for pepsinogen were significantly worse than random selection.

### 3.2 TST based on threshold values

#### 3.2.1 Varying initial pasture contamination

**Fig. 2:**
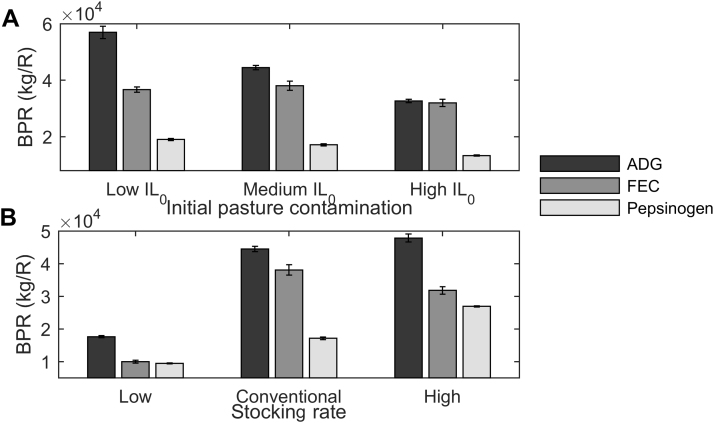
End of season (day 180) predictions for benefit per R (BPR), representing the benefit in empty bodyweight gain (kg) per change in frequency of resistant alleles amongst parasites (R), so the higher the value the more beneficial the strategy is perceived to be. Predictions are provided as an average of ten discrete populations of 500 calves simulated on A) pasture initially contaminated with 100, 200 or 500 L3/kg DM grass for calves kept at a stocking rate of 5 calves/ha or B) on pasture initially contaminated with 200 L3/kg DM grass an kept at stocking rates of 3, 5 or 7 calves/ha for groups of calves treated according to threshold values according to determinant criteria of average daily bodyweight gain (ADG, kg/d), faecal egg count (FEC, eggs/g) and pepsinogen.

Fig. 2A provides the BPR (kg/R) predictions for all levels of IL_0_ when treatments were administered according to threshold values for ADG, FEC or pepsinogen. For all determinant criteria, reducing IL_0_ led to an increased BPR; with the exception of FEC where the medium IL_0_ was predicted to have the greatest BPR. Using threshold values for ADG were predicted to result in the greatest BPR for all IL_0_, followed by FEC and finally pepsinogen. Whilst ADG threshold treatments were predicted to result in the greatest BPR, the relative advantage compared with FEC reduced as IL_0_ increased.

#### 3.2.2 Varying stocking rate

Fig. 2B provides the BPR (kg/R) predictions for all stocking rates when treatments were administered according to threshold values for ADG, FEC or pepsinogen. For all determinant criteria, increasing the stocking rate led to an increased BPR, with the exception of FEC where the conventional stocking rate was predicted to have the greatest BPR. Using threshold values for ADG were predicted to result in the greatest BPR for all stocking rates, followed by FEC and finally pepsinogen.

### 3.3 Comparison of TST strategies

For fixed percentage treatments, determinant criteria of pepsinogen and random selection were predicted to result in the greatest BPR for all IL_0_ and stocking rates (Fig. 1). For threshold treatments, the determinant criterion of ADG was predicted to result in the greatest BPR for all IL_0_ and stocking rates (Fig. 2). Further, treatments administered according to threshold values for ADG resulted in greater BPR predictions than when FEC or random selection were used to treat a fixed percentage of the herd. As such, treatments administered according to threshold values for ADG were identified as the best TST strategy as assessed by BPR.

## 4 Discussion

A previous simulation study (Berk et al., 2016c) used BPR predictions to assess both the method of implementing TST strategies (fixed percentage and threshold treatment) and various determinant criteria for treatment (ADG, FEC, pepsinogen or random selection), for a conventional stocking rate of 5 calves/ha and an IL_0_ of 200 infective L3 larvae/kg DM. That study concluded that treatment according to threshold values of ADG resulted in the greatest BPR; however, in the current study we hypothesised that variation in IL_0_ or stocking rate may be expected to impact upon recommendations for the best selection method for TST, or the best phenotypic trait on which to base treatment. As detailed in Section 3.3, treatments administered according to threshold values for ADG were identified as the best TST strategy (as assessed by BPR) for all levels of IL_0_ and stocking rate. As such, this reaffirms the conclusion/recommendation of the previous study (Berk et al., 2016c).

Whilst the conclusion of the present study ultimately remained the same as Berk et al. (2016c), the following trends were also predicted: 1) for all IL_0_, stocking rates and determinant criteria, decreasing the percentage of the herd treated increased BPR; 2) reducing IL_0_ increased BPR (with the exception of FEC threshold treatments); 3) for threshold treatments, the relative advantage of using ADG compared with FEC reduced as IL_0_ increased; 4) for fixed percentage treatments, BPR predictions were greatest at the conventional stocking rate, closely followed by the high stocking rate, with large reductions at the low stocking rate; 5) for threshold treatments, increased stocking rate increased BPR (with the exception of FEC threshold treatments).

The relationship between the percentage of the herd treated and BPR was previously identified in Berk et al. (2016c). Increasing the percentage of the herd treated resulted in an increased selective pressure for anthelmintic resistance (increased impact upon the frequency of R), which outweighed the relative advantage in weight gain, hence resulting in a reduced BPR. The relationship between IL_0_ and BPR was fairly consistent, with BPR decreasing as IL_0_ increased. High antigen exposure (high IL_0_) elicits greater density dependence effects, but also a faster development of acquired immunity in comparison to lower IL_0_ resulting in reduced pasture contamination through autoinfection in the latter parts of the grazing season (Berk et al., 2016b). This may be expected to result in a smaller *refugia* pool and consequently a greater impact of treatment upon the frequency of R, whilst the effects of IL_0_ on weight gain were minimal. When using threshold values of FEC, density-dependent effects on parasite fecundity obscured identification of calves with the highest worm burden. Density-dependent effects exert their greatest impact at high worm burdens (Michel et al., 1978); consequently, BPR predictions at low IL_0_ were worse than for medium and high IL_0_. Further, this also resulted in the apparent reduction in the relative advantage of using ADG compared with FEC as IL_0_ increased. With increasing IL_0_ the number of treatments administered according to ADG increased whereas those administered according to FEC decreased (Table 1).

**Table 1:** Total number of anthelmintic treatments administered to a population of 500 calves grazed on: 1) a pasture initially contaminated (IL_0_) with 100, 200 or 500 *Ostertagia ostertagi* L3/kg DM at stocking rate of 5 calves/ha and 2) at stocking rates of 3, 5 or 7 calves/ha and an IL_0_ of 200 *Ostertagia ostertagi* L3/kg DM. Average daily bodyweight gain (ADG, kg/d), faecal egg count (FEC, eggs/g DM) or pepsinogen (IUT/I) were used to determine which calves to treat according to either a fixed percentage or threshold values. Fixed percentage treatments were administered on 2 occasions, whilst assessments for threshold treatment were made every 3 weeks from 8 weeks post-turnout.

Method of selection		Fixed percentage		Threshold		
Percentage treated		10%	25%	–	–	–
Phenotypic trait		Any	Any	ADG	FEC	Pepsinogen
IL_0_ (L3/kg DM)	Stocking rate (calves/ha)					
100	5	100	250	784	134	471
200	3	100	250	753	185	185
200	5	100	250	795	99	474
200	7	100	250	976	133	568
500	5	100	250	844	81	496

For the relationship between stocking rate and BPR, higher stocking rates led to a greater number of parasitized calves excreting eggs onto pasture. The consequent increase in pasture contamination resulted in increased worm burdens and their associated impact upon weight gain (Fox, 1993). Thus, treatment had a greater potential to mitigate the impact of parasitism upon weight gain, thereby leading to increasing BPR predictions for increasing stocking rates. However, for determinant criterion FEC the threshold treatments were predicted to have the greatest BPR at a conventional stocking rate. At this stocking rate, Table 1 shows fewer calves exceeded the threshold values for FEC and were treated. Thus, reduced selective pressure for anthelmintic resistance, combined with large weight gain benefits for the most severely parasitized calves, led to increased BPR predictions at conventional stocking rates in comparison to high stocking rates. For fixed percentage treatments, conventional stocking rates were predicted to have a greater BPR than high stocking rates for all determinant criteria. In this instance, increasing stocking rates led to increasing pasture contamination (exposure) and an increased rate of immune acquisition (with associated reductions in weight gain). Hence, by the time assessment for treatment occurred there was less potential for weight gain improvements for the high stocking rate in comparison to the conventional stocking rate.

### 4.1 Perspectives

This study concluded that treatments administered according to threshold values for ADG were identified as the best TST strategy (as assessed by BPR) for all levels of IL_0_ and stocking rate. As such, this suggests that such factors do not need to be considered when designing appropriate TST strategies. However, it should be noted that the model of Berk et al. (2016c) made a number of assumptions in regards to the genetic mechanism of anthelmintic resistance (as described in section 2), which impact upon the rate at which resistance develops and consequently BPR predictions. In reality anthelmintic resistance is influenced by many factors, including: the number of genes and alleles involved (Barnes et al., 1995); the relative impact of resistant and susceptible alleles on efficacy and the persistence of activity; the mode of inheritance (Barnes et al., 1995); the level of pre-existing alleles; and potential reversion to susceptibility via resistant phenotype fitness disadvantages (Leathwick, 2013). Whilst these factors would be expected to alter BPR predictions, if applied equally across all treatment groups the same general principles and patterns would be expected to apply (Laurenson et al., 2016).

Further, the labour and financial costs of TST implementation need to be considered. For example, each phenotypic trait (on which to base treatment) has inherent costs associated with sampling and analysis (equipment and personnel); whilst threshold treatments require more frequent handling of cattle in comparison to fixed treatment percentages and consequently are more labour intensive. However, advances in precision agriculture, such as automated assessment of liveweight (Laca, 2009), may make the measurement of such traits more straightforward and perhaps less time consuming. There are several potential bottlenecks for the adoption of TST in livestock, one of which being the increased costs that might be associated with their implementation (Woodgate and Love, 2012, Charlier et al., 2014). This study provides strong support for the targeted control of parasitism; however, further work involving a cost-benefit analysis may prove useful in convincing cattle farmers of the long-term benefits of such TST strategies.
